# Synergistic effect and antibiofilm activity of the antimicrobial peptide K11 with conventional antibiotics against multidrug-resistant and extensively drug-resistant *Klebsiella pneumoniae*

**DOI:** 10.3389/fcimb.2023.1153868

**Published:** 2023-04-11

**Authors:** Chawalit Chatupheeraphat, Jiratchaya Peamchai, Sirirat Luk-in, Warawan Eiamphungporn

**Affiliations:** ^1^ Center for Research and Innovation, Faculty of Medical Technology, Mahidol University, Nakhon Pathom, Thailand; ^2^ Department of Clinical Microbiology and Applied Technology, Faculty of Medical Technology, Mahidol University, Bangkok, Thailand

**Keywords:** K11, multidrug-resistant *K. pneumoniae*, antibiofilm, synergy, meropenem, chloramphenicol, rifampicin

## Abstract

**Introduction:**

Infections caused by drug-resistant *Klebsiella pneumoniae* are now a serious problem for public health, associated with high morbidity and mortality due to limited treatment options. Therefore, new antibacterial agents or a combination of agents as the first line of treatment are urgently needed. K11 is a novel antimicrobial peptide (AMP) that has demonstrated *in vitro* antimicrobial activity against several types of bacteria. Additionally, K11 has previously shown no hemolytic activity. Herein, the antibacterial activity, the synergistic action of K11 in combination with different conventional antibiotics and the antibiofilm activity of K11 against multidrug-resistant (MDR) and extensively drug-resistant (XDR) *K. pneumoniae* were investigated. Meanwhile, the stability and ability to induce the bacterial resistance of K11 were also tested.

**Methods:**

Fifteen clinical isolates of MDR/XDR *K. pneumoniae* were used in this study. The minimum inhibitory concentration (MIC) of K11 against these isolates was determined by the broth microdilution method. *In vitro* synergy between K11 and antibiotics was evaluated using the checkerboard methodology. The antibiofilm activity of K11 against *K. pneumoniae* strong biofilm producers were explored by the crystal violet staining. The stability in different environments and resistance induction of K11 were evaluated by MIC determination.

**Results:**

The MIC values of K11 against MDR/XDR *K. pneumoniae* isolates were 8-512 μg/mL. Intriguingly, the synergistic effects were clearly observed for K11 in combination with chloramphenicol, meropenem, rifampicin, or ceftazidime, whereas no synergy was observed when K11 was combined with colistin. Besides, K11 effectively prevented biofilm formation against *K. pneumoniae* strong biofilm producers in a concentration-dependent manner starting at 0.25×MIC and exerted an enhancing effect when administered in combination with meropenem, chloramphenicol, or rifampicin. Additionally, K11 demonstrated high thermal and wide pH stability along with good stability in serum and physiological salts. Significantly, *K. pneumoniae* showed no induction of resistance even after prolonged exposure to a sub-inhibitory concentration of K11.

**Conclusion:**

These findings indicate that K11 is a promising candidate with potent antibacterial and antibiofilm activities without inducing resistance and acts synergistically with conventional antibiotics against drug-resistant *K. pneumoniae*.

## Introduction

The emergence of multidrug-resistant (MDR) and extensively drug-resistant (XDR) bacteria, combined with the failure of most current therapeutics and also a decline in new antibiotic development, poses a severe threat to global public health (Chinemerem Nwobodo et al., 2022; Collaborators AR, 2022). The infections caused by these bacteria result in rising morbidity and mortality rates and a burden on medical costs. It is estimated that worldwide, over 700,000 people die due to infections caused by drug-resist pathogens annually. Unless major actions are taken, this number is projected to rise to 10 million yearly by 2050 at an economic impact of 100 trillion dollars per year (O’Neill, 2014; Dadgostar, 2019). *Klebsiella pneumoniae* is a major drug-resistant pathogen associated with community-acquired (CA) and hospital-acquired (HA) infections. This pathogen is known to spread easily, and it is usually implicated in resistance to the highest-priority critically important antimicrobial agents (Holt et al., 2015; Marques et al., 2019). *K. pneumoniae* is one of the ‘ESKAPE’ pathogens that make up the most common antibiotic-resistant infections. MDR and XDR *K. pneumoniae* are among the most challenging infections to treat. The World Health Organization (WHO) recently published a global priority list of antibiotic-resistant bacteria, where carbapenem-resistant Enterobacteriaceae, including *K. pneumoniae*, was incorporated in the Priority 1 group (World Health Organization, 2017). Colistin (polymyxin E) was the most used antimicrobial against carbapenem-resistant Gram-negative bacteria. It has been considered a “last resort” antimicrobial to fight MDR bacterial infections (Li et al., 2006). Unfortunately, a gradual increase in the prevalence of colistin resistance has been documented in the last few years (Uzairue et al., 2022). The recent reports of colistin-resistant *K. pneumoniae* isolates raise concern, considering the further limitations of antimicrobial options and the high mortality rate associated with these infections. Thus, there is an urgency to develop new antimicrobial strategies to cope with these XDR pathogens. Intriguingly, *K. pneumoniae* can generate a thick layer of biofilm as one of its important virulence factors, enabling the bacteria to attach to living or abiotic surfaces, protecting antibiotic penetration, and reducing its effects (Vuotto et al., 2014). The resistance of biofilm-mediated infections to effective chemotherapy has an adverse impact on patient outcomes and survival. Considering the important role of biofilm formation for *K. pneumoniae* dissemination and virulence, finding alternative methods or new effective antimicrobial agents that can kill superbugs and inhibit biofilm formation is needed.

Antimicrobial peptides (AMPs) are promising candidates to overcome the above-mentioned drug-resistance crises. Several characteristics make them potential therapeutic alternatives to antibiotics. Obviously, the most important ones are the standard synthetic protocols, rapid killing kinetics, a broad range of antimicrobial action, and low potential for resistance development (Váradi et al., 2022). However, clinical applications of these AMPs have been hampered by several problems, such as cytotoxicity to host cells, low stability, and inactivity at physiological salt concentrations (Rios et al., 2016; Sarkar et al., 2021). Thus, the above obstacles have to be concerned when searching for or developing newly effective AMPs for therapeutic use. Additionally, a further aspect of the AMPs activity that has been much investigated in recent years and needs to be more deeply considered is their ability to affect biofilm formation. Many AMPs show antibiofilm activity against MDR bacteria, acting at different stages of biofilm formation, on disparate molecular targets and with various mechanisms (Di Somma et al., 2020). Another way to resolve the problem of drug-resistance bacteria is by combining different drugs. The effects of AMPs combined with antibiotics often exceed those of individual drugs. Therefore, the development of AMPs that have synergistic effects with antibiotics against MDR/XDR bacteria is important and challenging.

K11 is a synthetic peptide derived from natural AMPs (cecropin A1, melittin, and maganin 2), composed of 20 amino acids (KWKSFIKKLTKKFLHSAKKF-NH_2_). This peptide has been indicated as a promising candidate AMP with a good broad-spectrum antimicrobial effect, high therapeutic index, and no hemolytic activity (Jin-Jiang et al., 2012). Moreover, K11 has also been successfully used *in vivo* as a topic hydrogel solution against *Acinetobacter baumannii*-infected wounds (Rishi et al., 2018). However, there is no information on its *in vitro* antibacterial and antibiofilm activities against MDR/XDR Enterobacterales, especially *K. pneumoniae*. To the best of our knowledge, this is the first study to evaluate the antibacterial activity of K11 against MDR/XDR *K. pneumoniae* clinical isolates as well as to elucidate the antibiofilm potential of this peptide against MDR/XDR *K. pneumoniae* biofilms. Notably, the effect of K11 peptide in combination with conventional antibiotics used for treating bacteria and inhibiting biofilm was also determined. Besides antibacterial and antibiofilm activities, the characteristics of K11, including thermal and pH stability and stability in serum and under physiological salt conditions, were investigated. Additionally, the ability of *K. pneumoniae* standard strain to evolve resistance to K11 was monitored.

## Results

### Antibiotic susceptibility profiles of MDR/XDR *K. pneumoniae* clinical isolates

**Figure 1:**
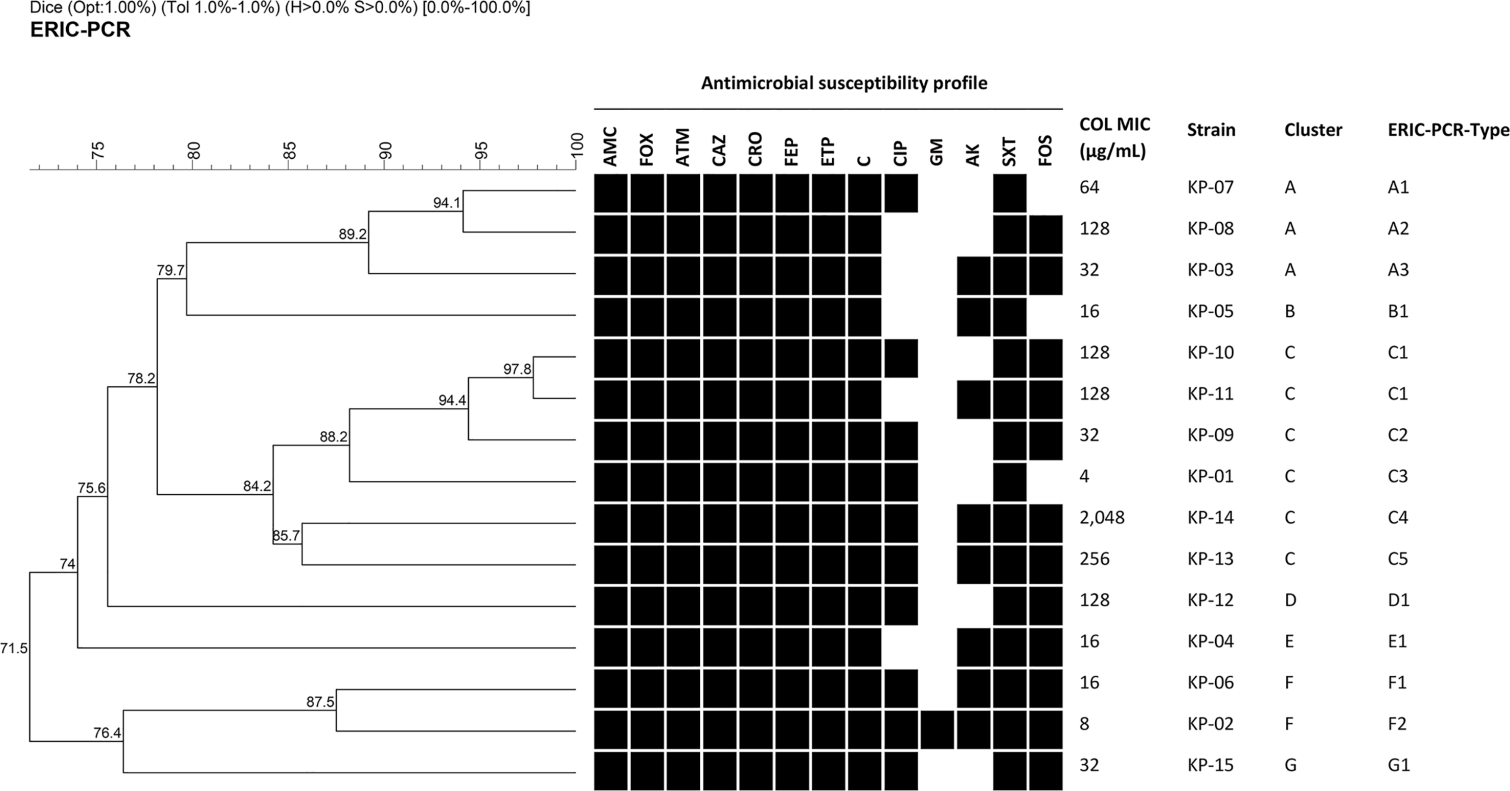
Dendrogram of genetic similarity generated by ERIC-PCR among fifteen MDR/XDR *K. pneumoniae* clinical isolates. Strain information showing antimicrobial susceptibility profiles, colistin MIC, cluster, and ERIC-PCR types. Black squares represent isolates that were resistant or intermediate to antimicrobial agents. Antimicrobial agents are abbreviated as follows: AMC, Amoxicillin/clavulanic acid; FOX, cefoxitin; ATM, aztreonam; CAZ, ceftazidime; CRO, ceftriaxone; FEP, cefepime; ETP, ertapenem; C, chloramphenicol; CIP, ciprofloxacin; GM, gentamicin; AK, amikacin; SXT, trimethoprim/sulfamethoxazole; FOS, fosfomycin; and COL, colistin.

Fifteen *K. pneumoniae* isolates demonstrated resistance to most antimicrobial agents (**Figure 1**). Notably, all isolates exhibited a resistant profile against amoxicillin/clavulanic acid (AMC), ceftazidime (CAZ), ceftriaxone (CRO), cefepime (FEP), cefoxitin (FOX), aztreonam (ATM), ertapenem (ETP), chloramphenicol (C) and trimethoprim/sulfamethoxazole (SXT). Additionally, all isolates were resistant to colistin (COL) with MICs ≥4 µg/mL. These results suggested that all isolates were deemed as MDR bacteria and some isolates were possibly XDR bacteria.

### Clonal relationship analysis

Among fifteen MDR/XDR *K. pneumoniae* clinical isolates, 7 clusters designated A to G and 14 different ERIC-PCR types were identified using a cut-off of 85% and 95% genetic similarity, respectively (**Figure 1**). Cluster C, the most common cluster, contained an ERIC-PCR type (C1) of two closely related patterns. However, all isolates had different antibiotic resistance profiles, as shown in **Figure 1**. Therefore, they were considered clonally unrelated strains, and none of the identical ERIC-PCR patterns was found among these isolates.

### Antibacterial activity of K11 against MDR/XDR *K. pneumoniae* isolates

MIC and MBC values of K11 were determined against *K. pneumoniae* reference strains and fifteen MDR/XDR *K. pneumoniae* clinical isolates. As shown in **Table 1**, K11 inhibited the growth of the reference strains and clinical isolates at concentrations of 2-4 μg/mL and 8-512 μg/mL, respectively. Moreover, the MBC values, which represented the bactericidal activity of K11, were identical to the MIC values or not greater than one dilution. Notably, the MIC and MBC values were higher against clinical isolates than reference strains. The MIC_50_ and MIC_90_ of K11 were 64 and 256 μg/mL, respectively.

**Table 1:** The MIC and MBC values of K11 against *K. pneumoniae* reference strains and MDR/XDR *K. pneumoniae* clinical isolates.

Organisms	MIC (µg/mL)	MBC (µg/mL)
*K. pneumoniae* ATCC BAA-1706	2	2
*K. pneumoniae* ATCC 70063	4	4
KP-01	8	8
KP-02	64	64
KP-03	16	16
KP-04	64	128
KP-05	8	8
KP-06	64	128
KP-07	16	16
KP-08	64	128
KP-09	64	128
KP-10	64	128
KP-11	64	64
KP-12	64	64
KP-13	32	32
KP-14	256	256
KP-15	512	512

### Synergy studies and checkerboard assay

The synergistic effects of the K11 combined with the antibiotics were determined as the fractional inhibitory concentration index (FICI). The results of FICI of K11 in combination with different antibiotics against all MDR/XDR *K. pneumoniae* clinical isolates are presented in **Table 2**. The combination of K11 and chloramphenicol exhibited a synergistic effect against 80% of all the tested isolates. Additionally, the combination of K11 and meropenem showed a synergistic effect against 73% of all isolates. These two combinations were the most promising of those tested against MDR/XDR *K. pneumoniae* isolates in this study. Of note, combining K11 and rifampicin also resulted in a synergistic effect against 67% of all isolates. Moreover, the combination of K11 and ceftazidime showed a synergistic effect against 53% of all isolates. Additionally, the combination of K11 and ciprofloxacin synergistically affected only 20% of the isolates, whereas the combination of K11 and colistin showed no synergistic effect on any tested bacterial isolates. No antagonism was observed in any combination. Notably, the synergistic effect of colistin and meropenem against MDR/XDR *K. pneumoniae* clinical isolates was also determined. Intriguingly, the synergistic effect of colistin and meropenem was observed against only 33% of all isolates as shown in **Table 2**.

**Table 2:** FICIs of K11 combined with conventional antibiotics to treat clinically isolated MDR/XDR *K. pneumoniae*.

Isolate	FICI^a^						FICI^a^
	K11						COL
	COL	MEM	C	RIF	CIP	CAZ	MEM
**KP-01**	0.625	0.25	0.375	1.0078	0.2578	0.5625	0.625
**KP-02**	1.0156	0.5	0.3125	0.2578	0.75	0.25	0.625
**KP-03**	1.0156	0.5	0.375	0.75	1.0313	1.0078	0.625
**KP-04**	1.0313	0.5313	0.5156	0.078	0.625	0.5078	0.75
**KP-05**	1.25	0.2813	1	0.375	1	1.0078	1
**KP-06**	1.0156	0.375	0.75	0.1328	0.625	0.1563	1.0078
**KP-07**	1.125	0.75	0.3125	0.5625	1	0.5078	0.5
**KP-08**	2.0156	0.375	0.3125	0.1563	0.75	0.5078	0.5
**KP-09**	0.5078	0.1875	0.1875	0.1328	0.5	0.3125	0.375
**KP-10**	0.502	0.375	0.25	0.56	0.375	0.1406	0.5
**KP-11**	1.0156	0.75	0.0938	0.125	0.5313	0.25	1
**KP-12**	1.0156	0.5	0.375	0.1406	1.0313	0.75	0.75
**KP-13**	2.0313	0.375	0.375	0.625	1.125	0.125	0.3125
**KP-14**	1	0.625	0.1328	0.0469	0.5156	0.5	1
**KP-15**	1.002	0.325	0.25	0.039	1.0313	0.5	0.625
**Synergy**	0%	(11) 73%	(12) 80%	(10) 67%	(3) 20%	(8) 53%	(5) 33%
**No interaction**	(15) 100%	(4) 27%	(3) 20%	(5) 33%	(12) 80%	(7) 47%	(10) 67%

^a^FICI is interpreted as synergy (FIC ≤0.5), no interaction (FIC>0.5-4), or antagonism (FIC ≥4).

^b^Antimicrobial agents are abbreviated as follows: ceftazidime (CAZ), chloramphenicol (C), ciprofloxacin (CIP), meropenem (MEM), rifampicin (RIF) and colistin (COL).

### Inhibition of biofilm formation

**Figure 2:**
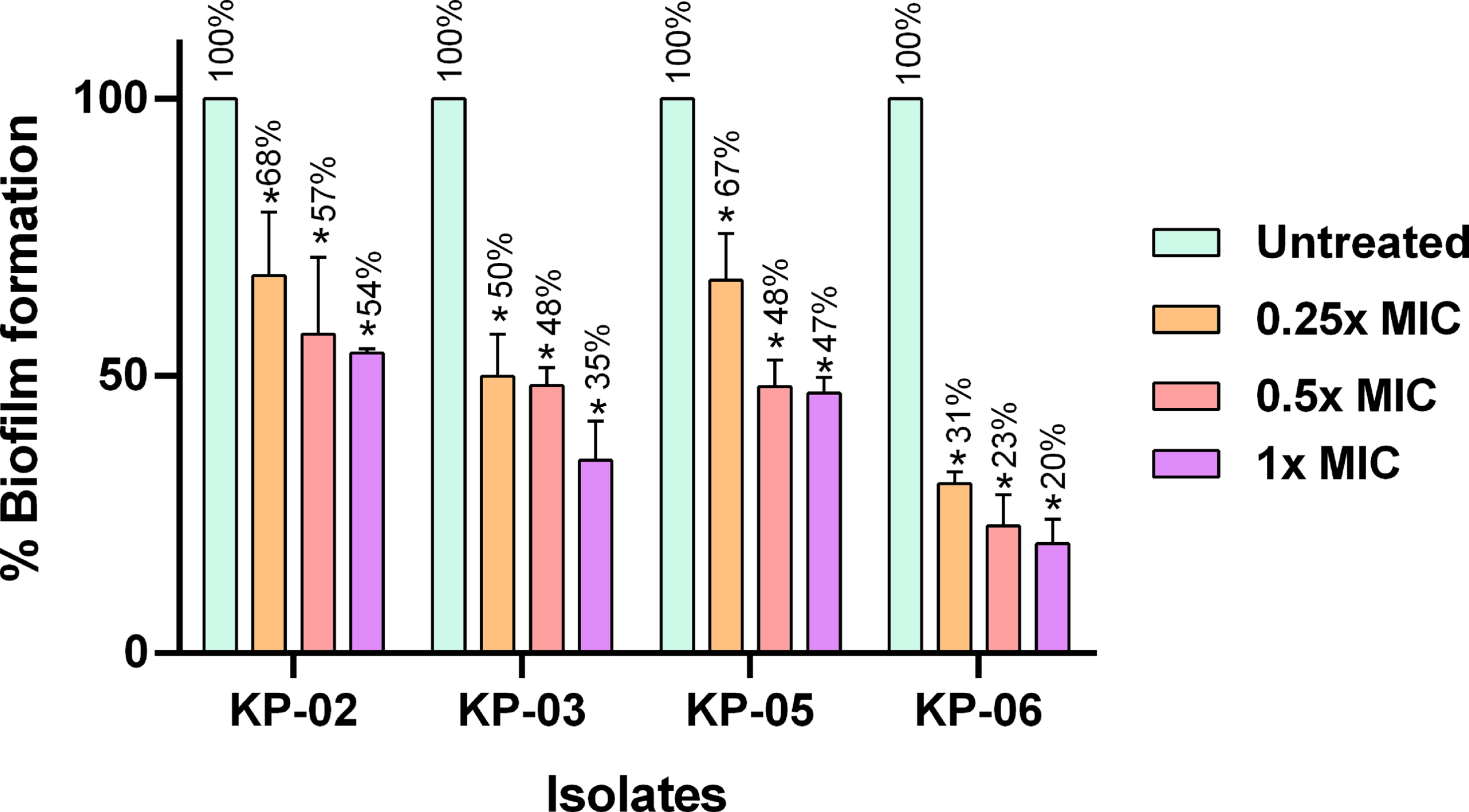
Inhibition of the growth of MDR/XDR *K. pneumoniae* biofilms. K11 was incubated with strong biofilm producers of *K. pneumoniae* at 0.25×, 0.5×, and 1×MIC of the peptide. The untreated bar indicates MDR/XDR *K. pneumoniae* biofilm without any antimicrobial agent. Crystal violet was used to determine the total biomass of the biofilm. The data were analyzed by one-way ANOVA; *P value <0.05. Percent numbers indicate % biofilm formation.

The inhibitory effect of K11 on biofilm formation by strong-biofilm producers of MDR/XDR *K. pneumoniae* was studied. The results of the antibiofilm efficacy of K11 at different concentrations on initial biofilm formation are shown in **Figure 2**. Compared with untreated controls, the different concentrations of K11 exhibited dose-dependent biofilm inhibition against each of four strong-biofilm-forming strains of MDR/XDR *K. pneumoniae* at 24 h. It was demonstrated that K11 caused approximately 32% to 80% decrease in the biofilm biomass at the concentration of 2 up to 64 μg/mL (0.25×MIC to 1×MIC). Significant biofilm inhibition was observed at especially 0.25×, 0.5× and 1×MIC values of K11.

### Effects of K11 combined with antibiotics on MDR/XDR *K. pneumoniae* biofilm

**Figure 3:**
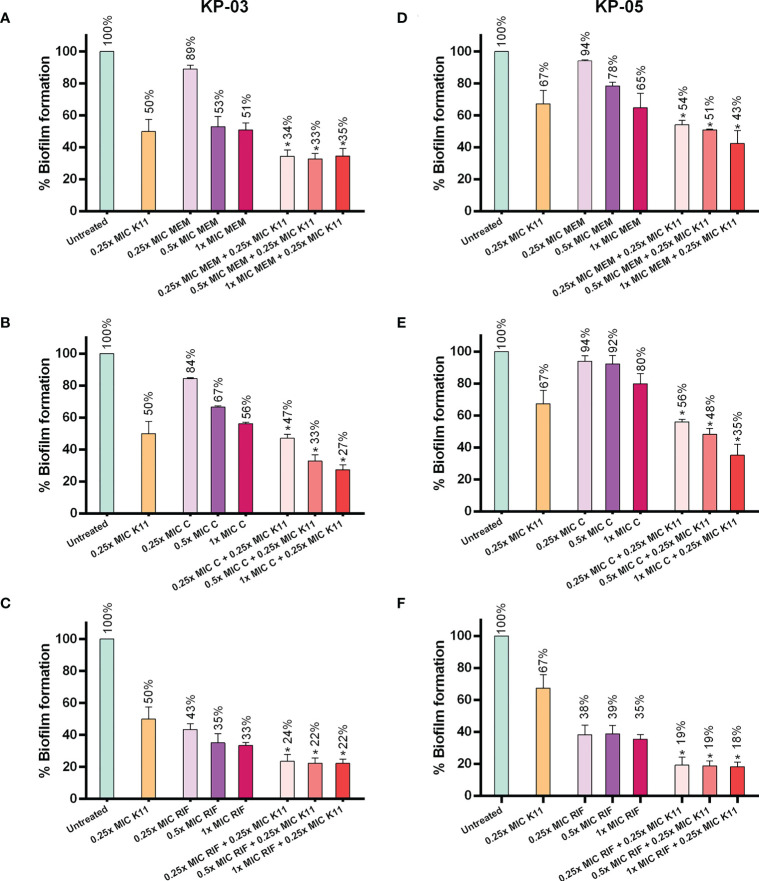
Effects of K11 against MDR/XDR *K. pneumoniae* biofilm when administered in combination with conventional antibiotics. Two representatives of MDR/XDR-strong biofilm producers of *K. pneumoniae*, KP-03 and KP-05, were tested with K11 or antibiotic alone or in the combination of K11 and antibiotics for biofilm-inhibiting activity. **(A–C)** KP-03 isolate was tested with K11 or antibiotic; meropenem (MEM), chloramphenicol (C), rifampicin (RIF) alone or in the combination of K11 and antibiotics. **(D–F)** KP-05 isolate was tested with K11 or antibiotic; meropenem (MEM), chloramphenicol (C), rifampicin (RIF)) alone or in the combination with K11 and antibiotics. The untreated bar indicates MDR/XDR *K. pneumoniae* biofilm without any antimicrobial agent. Crystal violet was used to determine the total biomass of the biofilm. The data was calculated by one-way ANOVA; *P value <0.05. Percent numbers indicate % biofilm formation.

The effects of K11 in combination with conventional antibiotics on biofilm inhibition were investigated (**Figure 3**). In this experiment, K11 concentration was fixed at 0.25×MIC, while the concentrations of antibiotics were varied at 0.25×, 0.5×, and 1×MIC. The MIC of meropenem, chloramphenicol, and rifampicin were similar against two representatives of MDR/XDR-strong biofilm producers of *K. pneumoniae* as 64, 16, and 32 µg/mL, respectively. The results showed that K11, combined with meropenem, chloramphenicol, or rifampicin, enhanced activity against two MDR/XDR-strong biofilm producers of *K. pneumoniae*. Of note, when K11 was combined with a particular antibiotic, the efficiency in inhibiting biofilm formation was significantly higher than those obtained with K11 or antibiotic alone. These findings indicated that the biofilm-inhibiting activity of K11 against MDR/XDR *K. pneumoniae* could be augmented by combination with antibiotics.

### Thermal and pH stabilities

The stability of AMPs is crucial for storage and applications. Different temperatures and pH environments might influence the sensitivity of bacteria to some peptides. Hence, the thermal- and pH-resistant stabilities of K11 were evaluated by exposure to different temperatures and pH buffers for 1 h. Interestingly, K11 maintained its antimicrobial activity against *K. pneumoniae* ATCC 70063 and *K. pneumoniae* ATCC BAA-1706 when treated at 50, 70, or 90°C (**Table 3**). Furthermore, K11 also exhibited similar antimicrobial activity against the reference strains over a relatively wide pH range from 2.0 to 10.0 (**Table 4**). These results revealed that K11 had excellent thermal- and pH-resistant stabilities.

**Table 3:** The MIC values of the K11 against *K. pneumoniae* reference strains after different temperature treatments.

Bacterial Strains	Temperature (°C)	Antimicrobial activity MIC (µg/mL)
*K. pneumoniae* ATCC BAA-1706	25 (control)	2
	37	2
	50	2
	70	2
	90	2
*K. pneumoniae* ATCC 70063	25 (control)	4
	37	4
	50	4
	70	4
	90	4

**Table 4:** The MIC values of the K11 against *K. pneumoniae* reference strains after different pH treatments.

Bacterial Strains	pH	Antimicrobial activity MIC (µg/mL)
*K. pneumoniae* ATCC BAA-1706	7 (control)	2
	2	2
	4	2
	6	2
	8	2
	10	2
*K. pneumoniae* ATCC 70063	7 (control)	4
	2	4
	4	4
	6	4
	8	4
	10	4

### Salt and serum stabilities

The sensitivity of AMPs to physiological salts and serum has been regarded as an inevitable problem in clinical applications. Therefore, the salt- and serum-resistant stabilities of K11 were further assessed by exposure to different physiological salts and fetal bovine serum (FBS) concentrations. The results showed that treatment with various concentrations of NaCl, MgCl_2_, and FeCl_3_ had no apparent influence on the antimicrobial activity of K11 against tested strains (**Table 5**). The ability of K11 to inhibit bacterial growth was not significantly decreased even at final concentrations of salts. Additionally, K11 did not exhibit any change in MIC after treatment in 25% and 50% FBS for 1 h (**Table 6**). These results indicated that K11 showed good resistance to physiological salts, such as NaCl, MgCl_2_, and FeCl_3_, as well as serum.

**Table 5:** The MIC values of the K11 against *K. pneumoniae* reference strains in the presence of physiological salts.

Bacterial Strains	Salt	Concentration	Antimicrobial activity MIC (µg/mL)
*K. pneumoniae* ATCC BAA-1706	Control	0	2
	NaCl	100 mM	2
		150mM	4
		200 mM	4
	MgCl_2_	0.5 mM	2
		1 mM	2
		2 mM	2
	FeCl_3_	1 µM	2
		4 µM	2
		8 µM	2
*K. pneumoniae* ATCC 70063	Control	0	4
	NaCl	100 mM	4
		150 mM	4
		200 mM	4
	MgCl_2_	0.5 mM	4
		1 mM	4
		2 mM	8
	FeCl_3_	1 µM	4
		4 µM	4
		8 µM	4

**Table 6:** The MIC values of the K11 against *K. pneumoniae* reference strains after FBS treatment.

Bacterial Strains	%FBS	Antimicrobial activity MIC (µg/mL)
*K. pneumoniae* ATCC BAA-1706	0 (control)	2
	25	2
	50	2
*K. pneumoniae* ATCC 70063	0 (control)	4
	25	4
	50	4

### Resistance induction by serial passages

**Figure 4:**
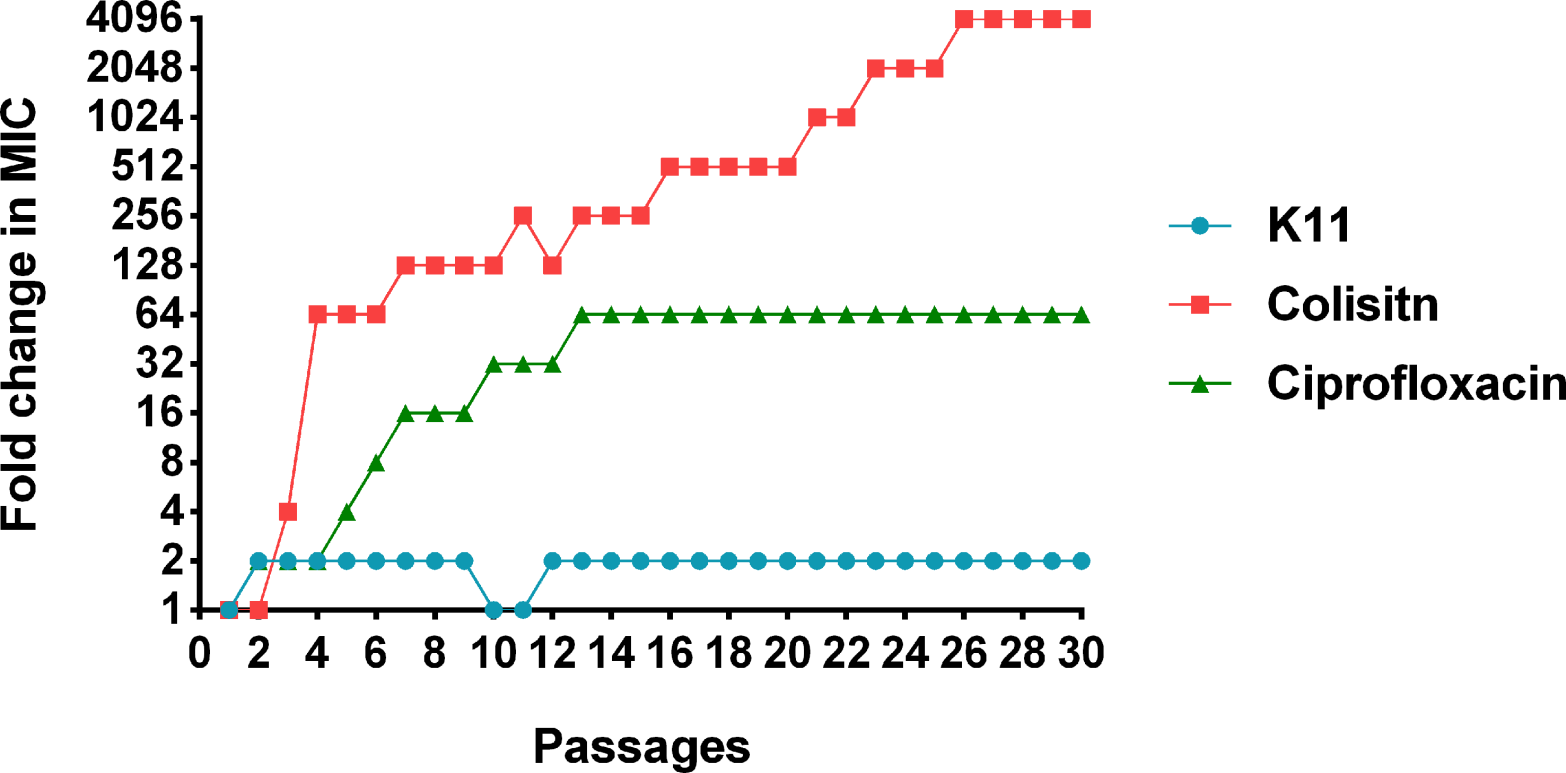
The change of MICs of K11 against *K. pneumoniae* ATCC BAA-1706 strain after 30 consecutive days of treatment. Ciprofloxacin and colistin were used as controls.

The tendencies of antimicrobial resistance induced by ciprofloxacin, colistin, and K11 against *K. pneumoniae* ATCC BAA-1706 reference strain was monitored by determining the MIC values of drugs and peptide after 30 days of consecutive treatments of bacteria at the concentration of 0.5×MIC. The MIC of ciprofloxacin significantly increased to 16-fold after 7 serial passages and reached 64-fold after 12 passages, then maintained this fold change until 30 passages (**Figure 4**). Noteworthy, the MIC of colistin remarkably increased to 64-fold after a few passages and continue to rise dramatically over 30 passages (4,096-fold). Fortunately, slight variations in the MIC of K11 were observed over 30 passages, and the MIC did not rise above 2-fold. This result suggested that K11 showed a negligible drug resistance induction tendency.

## Discussion

The emergence of drug-resistant pathogens is increasing, and the capacity of currently available antibiotics to control bacterial infections is declining. Currently, AMPs have gained increasing attention as potential novel antimicrobial alternatives for combating infections caused by conventional antibiotic-resistant bacteria and/or associated with biofilm. In this study, the *in vitro* antimicrobial and antibiofilm activities of K11, alone or in combinations with different conventional antibiotics against MDR/XDR *K. pneumoniae* clinical isolates were investigated. All tested *K. pneumoniae* isolates included in our study exhibited a resistant phenotype toward major classes of antibiotics that were defined as MDR, and some of these were XDR according to international standard definitions for acquired resistance (Magiorakos et al., 2012). ERIC-PCR fingerprint was used to determine the genetic variation among strains. Notably, most isolates were clonally unrelated strains, and none of the identical ERIC-PCR patterns was found among these isolates. Our results demonstrated high diversity among tested MDR/XDR *K. pneumoniae* strains, which might be desirable since these strains could be good representatives for the determination of the antimicrobial activity of K11.

K11 is a synthetic peptide with remarkable antibacterial properties for many bacteria and its optimal therapeutic index. It has been elucidated that K11 bound and twisted in the membrane, causing an effective membrane disruption, potentially leading to bacterial death (Ramos-Martín et al., 2020). It has been previously shown that K11 exhibited antibacterial activity against clinically isolated *K. pneumoniae* with MICs 0.5-4 µg/mL (Jin-Jiang et al., 2012). This result is consistent with our result which the MICs of K11 against *K. pneumoniae* reference strains were 2-4 µg/mL. However, our study revealed the higher MICs of K11 against MDR/XDR *K. pneumoniae* that were 8-512 µg/mL. The explanation for this appearance may be possibly cross-resistance between colistin and K11 since all our strains were colistin-resistant. Previous studies revealed that the cross-resistance between colistin and other AMPs could exist since they share similar membrane-binding mechanisms (Napier et al., 2013; Al-Farsi et al., 2019). Intriguingly, the MBC values of K11 showed proximity to those of MIC, which indicated the bactericidal effect of K11.

One plausible strategy to treat drug-resistant-associated infections is using a combination of antimicrobial agents. In the literature, using AMPs in conjunction with other antibiotics improved the antimicrobial efficacy against drug-resistant bacteria compared with each individual. Several interesting findings of the previous studies include the synergistic effects of P10 and ceftazidime or doripenem, as well as nisin and colistin against XDR *A. baumannii* and colistin-resistant *P. aeruginosa* (Jahangiri et al., 2021), the synergistic effects of SET-M33 and rifampicin, aztreonam, or meropenem against MDR and XDR isolates of *P. aeruginosa*, *A. baumannii* and *K. pneumoniae* (Pollini et al., 2017), the synergistic activity of S-thanatin and cefepime against MDR *K. pneumoniae* (Wu et al., 2009) and the synergy between melittin and doripenem or ceftazidime against MDR isolates of *A. baumannii* and *P. aeruginosa* (Akbari et al., 2019). In our study, although colistin seemed to confer cross-resistance to K11, as observed by relatively high MICs against MDR/XDR *K. pneumoniae* isolates when compared with reference strains, K11 exerted a remarkably synergistic effect when combined with several conventional antibiotics. The combined effect of this peptide has not been investigated before with any other antimicrobial agent. Among various antibiotic combinations, K11 exhibited mostly synergistic effects with chloramphenicol, meropenem, rifampicin, and ceftazidime against tested isolates. Previously, other AMPs were reported synergistic effects in combination with β-lactams and rifampicin (Wu et al., 2009; Soren et al., 2015; Pollini et al., 2017; Akbari et al., 2019; Jahangiri et al., 2021). However, the related information on the synergy between AMPs and chloramphenicol was more limited, but a few studies presented the synergistic effects between chloramphenicol and polymyxin B, which is also a cationic AMP (Abdul Rahim et al., 2015; Abdul Rahim et al., 2021). The mechanisms behind these synergies must be further elucidated; nevertheless, hypothetically, it is presumably that K11 can increase the membrane permeability allowing the entrance of the antibiotic, such as chloramphenicol and rifampicin into the cells. Moreover, the synergy with meropenem or ceftazidime could be due to the combined effect of both K11 and β-lactams on the cell envelope. Notably, since the MIC of K11, when in combination, was lowered itself, it might suggest that the antibiotic may also potentiate another mechanism of action of K11, probably at the cytoplasmic level. Additionally, the synergistic effect of colistin and meropenem against MDR/XDR *K. pneumoniae* clinical isolates was evaluated since colistin-carbapenem combination therapy has been used widely for the treatment of drug-resistant Gram-negative pathogens (Papst et al., 2018). Interestingly, the less synergistic effect between colistin and meropenem against all isolates was shown. It has been previously reported that the combination of meropenem with polymyxins against *K. pneumoniae* exhibited an *in vitro* synergy rate of 34% (Zusman et al., 2013). This synergy rate is in good agreement with the rate in our study (33%). Profitably, our data indicated that K11 was more synergistic than colistin when combined with meropenem. Noteworthy, no synergy was observed when combining K11 with colistin. This result supported the notion that there was a cross-resistance between colistin and K11, which probably exerted a similar mechanism or influenced the same target against drug-resistant bacteria.

AMPs have emerged as promising alternatives to conventional antibiotics for treating persistent infections caused by biofilm. Recently, growing interest has been devoted to the possible use of AMPs as antibiofilm agents (Mansour et al., 2015; Batoni et al., 2016). Herein, K11 was first demonstrated the antibiofilm activity against the strong biofilm-producing MDR/XDR-*K. pneumoniae* isolates. Our results showed that using K11 alone at 0.25×MIC significantly reduced the biofilm formation and the activity was increased when increasing the concentration of K11 represented in a concentration-dependent manner. Several reports demonstrated that AMPs could inhibit the formation of biofilm at a concentration lower than MIC which indicated the high potency of AMPs in biofilm inhibition (de la Fuente-Núñez et al., 2015; Moazzezy et al., 2020; Galdiero et al., 2021; Ajish et al., 2022). Furthermore, K11, combined with meropenem, chloramphenicol, or rifampicin, showed enhancing effects against representative biofilm-forming *K. pneumoniae*. Intriguingly, the combination of K11 with antibiotics was more effective than K11 or antibiotics alone. Our findings are consistent with other studies reporting that the biofilm-inhibiting activity of AMPs against MDR *K. pneumoniae* could be enhanced by combination with conventional antibiotics (Ribeiro et al., 2015; Duan et al., 2021). The possible mechanisms of AMPs to potentiate the antibiofilm activity when combined with antibiotics were reported, such as AMPs promoting antibiotic uptake and interfering with signaling pathways involved in biofilm formation (Grassi et al., 2017). Regarding our results, K11 may be useful not only as a compound for developing novel antimicrobials but also as a compound that may counteract bacterial biofilm formation.

One of the many hindrances to developing AMPs for clinical applications is their significantly reduced antimicrobial potency due to low stability in certain environments (Rollema et al., 1995; Abou Alaiwa et al., 2014; Zong et al., 2016; Chen et al., 2020). The pH and thermal stability of AMPs are essential for their preparation, processing, and storage (Zong et al., 2016). At the same time, the stability of AMPs in serum and under physiological salt conditions is also crucial when administrating AMPs *in vivo* (Rodríguez et al., 2021). Hence, the stability of K11 in various environments was studied. Our results demonstrated that K11 maintained antimicrobial activity in extreme temperatures and an acid-base environment, as well as in the physiological salts and serum environment. To the best of our knowledge, this is the first time that the admirable stability of K11 was elucidated. In general, the cytotoxicity of AMPs against eukaryotic cells is one of the significant problems in their clinical application. Of note, a previous study showed that K11 had low hemolytic activity and a high therapeutic index (Jin-Jiang et al., 2012). Hemolytic activity is traditionally indicated for toxicity in preclinical studies. No significant hemolysis was observed for K11, up to 500 μg/mL (Jin-Jiang et al., 2012), while more than 100 μg/mL of colistin showed significant hemolytic activity (Witherell et al., 2021). Interestingly, K11 has been shown that it causes no cytotoxicity using a murine excision model (Rishi et al., 2018). Accordingly, these findings indicated that K11 would be a potential candidate for treating drug-resistant *K. pneumoniae* infection. However, further study on its potency and stability *in vivo* still is needed.

Although AMPs are promising candidates for the development of novel alternative antibiotics, many studies have shown that some bacteria are resistant to certain AMPs (Anaya-López et al., 2013; Joo et al., 2016). In this study, K11 was tested against a reference strain of *K. pneumoniae* in a serial passage experiment to induce bacterial resistance. Ciprofloxacin and colistin were used as comparators of K11. Interestingly, the development of resistance to K11 by *K. pneumoniae* ATCC BAA-1706 was trivial compared to that generated by ciprofloxacin and colistin in the tested conditions. Significantly, high resistance to colistin was induced through short-time exposure. Our results suggested that it was difficult for K11 to induce bacterial resistance when compared with conventional antibiotics, consistent with several previous studies examining other AMPs (Hong et al., 2012; Pollard et al., 2012; Dolzani et al., 2019; Zhou et al., 2021). Altogether, considering the interest in using AMPs clinically to treat MDR/XDR-*K. pneumoniae* infection, K11 appears to be a possible alternative for use.

In summary, K11 may serve as a potential AMP for the treatment of MDR/XDR-*K. pneumoniae* infection. It is a short peptide that is simple to produce with a high product yield, highlighting it as a promising candidate. Aside from the previously reported antimicrobial activity of K11, its ability to synergize with different antibiotics against MDR/XDR-*K. pneumoniae* isolates were disclosed in this study. Additionally, K11 significantly prevented biofilm formation when used alone and in combination with other antibiotics. K11 also exhibited high thermal and wide pH stability, and great stability in the presence of serum and physiological salts. Furthermore, K11 was quite unlikely to induce bacterial resistance. These findings are important for developing new therapeutic regimens to treat MDR/XDR *K. pneumoniae* infections.

## Materials and methods

### Bacterial strains

In this study, fifteen non-duplicate clinical isolates of MDR/XDR *K. pneumoniae* were obtained from the bacterial repository of the Division of Infectious Diseases and Tropical Medicine, Department of Medicine, Faculty of Medicine Siriraj Hospital, Mahidol University. The species were confirmed using standard biochemical tests. Antimicrobial susceptibility testing was also carried out by the disk diffusion method according to CLSI guidelines (Clinical and Laboratory Standards Institute, 2021). Furthermore, all isolates were tested for colistin resistance by broth microdilution based on CLSI recommendations. Following the CLSI breakpoints, colistin resistance was defined as a MIC of ≥4 µg/mL (Clinical and Laboratory Standards Institute, 2021). The MDR bacteria was defined as bacteria that are resistant to at least one agent in three or more antimicrobial categories, while the XDR bacteria refer to bacteria that are resistant to at least one agent in all but two or fewer antimicrobial categories based on laboratory results. *E. coli* ATCC 25922 was used as a reference control strain for susceptibility testing. Other reference strains, including *K. pneumoniae* ATCC 70063 and *K. pneumoniae* ATCC BAA-1706 (*bla*_KPC_ negative), were used as control strains throughout the study.

### Clonal relationship analysis

The clonal relatedness of all *K. pneumoniae* clinical isolates was determined by enterobacterial repetitive intergenic consensus PCR (ERIC-PCR) using primers, ERIC-1 (5’-ATGTAAGCTCCTGGGGATTCAC-3’) and ERIC-2 (5’-AAGTAAGTGACTGGGGTGAGCG-3’) as those previously described (Versalovic et al., 1991). The genomic DNA of all isolates was extracted using a TIANamp bacteria DNA kit (Tiangen Biotech, Beijing, China) according to the manufacturer’s protocol. The PCR amplification was performed with slight modification as follows: initial denaturation at 94°C for 5min, followed by 35 cycles of denaturation at 94°C for 1 min, annealing at 38°C for 1 min, and extension at 72°C for 3 min, with a final extension at 72°C for 10 min. ERIC-PCR patterns were compared by InfoQuest™FP Software, version 4.5 (Bio-Rad, Hercules, USA) using the Dice coefficient. The dendrogram was generated by the unweighted pair group method with arithmetic means (UPGMA) using 1.0% optimization and 1.0% band position tolerance. Percentage similarities were used to assess the relatedness of the clones (Widerström et al., 2007).

### Peptide synthesis and antibiotics

K11 with C-terminal amidation was synthesized by GenScript (Piscataway, NJ, USA) using solid-phase Fmoc chemistry and purified to >90% purity using HPLC. The mass was confirmed by MALDI-TOF mass spectroscopy. The antibiotics were acquired from TCI (Tokyo, Japan), except colistin sulfate, which was obtained from Chem-Impex Int’l Inc. (Wood Dale, IL, USA). Antibiotic disks were purchased from Oxoid (Oxoid Ltd., UK).

### Minimum inhibitory concentration and minimum bactericidal concentration determination

The ability of K11 to inhibit bacterial growth was determined by the broth microdilution method (Clinical and Laboratory Standards Institute, 2018). Briefly, a series of 2-fold dilutions of K11 were prepared from 1,024 to 2 µg/mL in cation-adjusted Mueller-Hinton broth (CAMHB), Then 50 µL of each peptide dilution was added to each well of polystyrene 96-well U-bottom microplates. The log-phase cultures were adjusted to 0.5 McFarland standard and diluted 1:100 in CAMHB to provide the density of 10^6^ CFU/mL. Consequently, 50 µL of diluted bacterial suspension was added to wells containing 50 µL of peptide solution, followed by incubation for 20-24 h at 37°C. The lowest concentration of peptide that completely inhibited bacterial growth was regarded to be the MIC. To evaluate MBC, a volume of 10 μL was taken from each well with no visible growth, inoculated on Mueller-Hinton agar (MHA) plates, and incubated at 37°C. The MBC was defined as the lowest concentration of antimicrobials that killed at least 99.9% of the initial inoculums (Clinical and Laboratory Standards Institute, 1999).

### Synergy studies and checkerboard assay

The synergistic effects of K11 in combination with conventional antibiotics, including meropenem, chloramphenicol, rifampicin, colistin, ciprofloxacin, and ceftazidime, were assessed using the checkerboard method (Zhang et al., 2019). Briefly, K11 and antibiotics were prepared at 4×of desired concentrations by a two-fold dilution method. Next, 25 µL of K11 and antibiotic dilutions with different concentrations were added to the designed wells on the plate to obtain different proportions with K11 or antibiotic concentrations. Bacterial cultures were grown to log phase and diluted to 10^6^ CFU/mL in CAMHB. Then, 50 µL of diluted bacterial suspension was added to each dilution of K11 and antibiotic combinations to give the final desired concentrations and subsequently incubated for 20-24 h at 37°C. The FICI values were calculated using the concentration combinations with the highest combination effects: FICI = MIC of drug A in combination/MIC of drug A alone + MIC of drug B in combination/MIC of drug B alone. The antimicrobial combination was defined as synergy when the FICI was ≤ 0.5, no interaction when 0.5 <FICI <4, and antagonism when the FICI was ≥ 4.

### Inhibition of biofilm formation

The ability of K11 to inhibit biofilm formation against four representatives of strong biofilm-producing MDR/XDR *K. pneumoniae* was examined by the crystal violet staining as previously described with few modifications (Gopal et al., 2014; Liu et al., 2022). Overnight cultures of bacteria were conducted at 37°C in CAMHB. Subsequently, cultures were diluted to 10^8^ CFU/mL in CAMHB supplemented with 0.5% glucose. A 50 µL aliquot of bacterial suspension was transferred to the wells of a sterile polystyrene 96-well flat-bottom plate. The stock solutions (0.5×, 1×, and 2×MIC) of K11 were prepared, adding 50 µL of each stock solution to the wells to a final concentration of 0.25×, 0.5×, and 1×MIC of the peptide. The mixtures were incubated for 24 h at 37°C to allow biofilm formation. After incubation, the medium was discarded from the wells, and any free-floating planktonic cells were removed by washing the wells with sterile deionized water. The biofilms were fixed with 100% methanol for 10 min, and then, the plates were allowed to dry. After that, wells were stained with 1% (w/v) crystal violet for 30 min. The excess stain was then thoroughly rinsed away with deionized water, and the plates were left to dry. Once dry, 95% ethanol was added into wells to solubilize the stain. The optical density (OD) was measured at 595 nm. The percentage of biofilm formation was calculated based on the growth control.

### Inhibition of biofilm formation by combined K11 and antibiotics

The antibiofilm efficiency of combination K11 and meropenem, chloramphenicol, or rifampicin was conducted by crystal violet assay as described above. Two strong biofilm-producer strains of MDR/XDR *K. pneumoniae* were selected for this study. The concentrations of 0.25×, 0.5×, and 1×MIC of antibiotics were tested in the presence of a fixed concentration of peptide (0.25×MIC).

### Thermal and pH stability assays

The temperature and pH stabilities of K11 were evaluated by the MIC assay, as mentioned above. To assess the thermal stability, Aliquots of 2 mg/mL of K11 in sterile deionized water were incubated for 1 h at 25°C, 37°C, 50°C, 70°C, and 90°C. Then, a series of 2-fold dilutions of K11 were prepared in CAMHB, 50 µL of each peptide solution was transferred to the well of a 96-well microplate. An equal volume of *K. pneumoniae* ATCC 70063 or *K. pneumoniae* ATCC BAA-1706 (10^6^ CFU/mL) suspension was added, and the mixture was incubated at 37°C for 20-24 h (Li et al., 2021). To examine the pH stability, the pH was adjusted to range from 2.0 to 10.0 with the following 100 mM buffers: glycine-HCl buffer (pH 2.0), sodium acetate buffer (pH 4.0), sodium phosphate buffer (pH 6.0), Tris-HCl buffer (pH 8.0), and glycine-NaOH buffer (pH 10.0). Then, 2 mg/mL of K11 was prepared in buffers with different pH values (pH 2.0-10.0) and incubated for 1 h at 37°C. Afterward, the pH of the reaction solution was neutralized by HCl or NaOH to terminate the reaction. The serially diluted peptide solutions were conducted, then treated against the tested strains, and the mixtures were incubated at 37°C for 20-24 h (Cao et al., 2015; Zong et al., 2016). The MICs were observed.

### Salt and serum stability assays

The effects of various cations and serum on the antimicrobial activity of K11 were investigated against *K. pneumoniae* ATCC 70063 and *K. pneumoniae* ATCC BAA-1706 by the MIC assay, as described above. For salt stability, 2 mg/mL of K11 was dissolved in solutions containing different salts at their physiological concentrations; NaCl (100, 150, and 200 mM), MgCl_2_ (0.5, 1 and 2 mM), and FeCl_3_ (1, 4, and 8 mM), then incubated for 1 h at 37°C. Subsequently, serially 2-fold diluted K11 solutions were prepared in CAMHB, and aliquots of each peptide solution were added to a 96-well plate. An equal volume of bacterial suspension was added, and the mixtures were incubated at 37°C for 20-24 h (Ko et al., 2019). For serum stability, 2 mg/mL of K11 was prepared in solution with 25% and 50% FBS and incubated for 1 h at 37°C, then heat activated for 30 min at 60°C. A series of 2-fold dilutions of K11 were performed in CAMHB and transferred to a 96-well plate. The diluted bacterial suspension was added to the well, and mixtures were incubated for 20-24 h at 37°C (Oliva et al., 2018). The antimicrobial activity was determined by measuring the MICs.

### Resistance induction by serial passages

The resistance development of K11, ciprofloxacin, and colistin was assessed according to the previously described protocol (Pollard et al., 2012). Briefly, MIC values for a *K. pneumoniae* ATCC BAA-1706 were first examined and recorded. After incubation at 37°C for 20-24 h, serial passaging was initiated by harvesting bacterial cells growing at 0.5×MIC values and inoculating them into fresh CAMHB. This inoculum was subjected to another MIC assay. After a 20-24 h incubation period, cells growing at 0.5×MIC from the previous passage were once again harvested and assayed for the MIC. The process was repeated for 30 passages. The MIC values for K11 and antibiotics were recorded and plotted as the MIC fold change compared to day 1 of the experiment.

### Statistical analysis

All experiments were performed with at least two reproductions with no less than two replicates each. The quantitative data are presented as mean ± standard deviation (SD). The results were analyzed for normal distribution using the Shapiro-Wilk test (P value <0.05). When no significant difference from normality was identified, the significance of differences among groups was analyzed by one-way analysis of variance (ANOVA). All statistical calculations were performed using GraphPad Prism version 8.4.3. Values of P <0.05 were considered statistically significant.

## Data availability statement

The original contributions presented in the study are included in the article/supplementary material. Further inquiries can be directed to the corresponding author.

## Author contributions

CC, SL-I, and WE contributed to the design of the experiments. CC, and JP performed the experiments. CC and JP wrote the initial draft of the manuscript. CC, JP, SL-I and WE contributed to the acquisition, analysis, and interpretation of the data included in this manuscript. SL-I and WE revised the manuscript. All authors contributed to the article and approved the submitted version.
